# Demonstrating clean energy transition scenarios in sector-coupled and renewable-based energy communities

**DOI:** 10.12688/openreseurope.16693.2

**Published:** 2024-09-02

**Authors:** Md Nasimul Islam Maruf, Shadman Mahmud, Iván S. Pasarín, Federico Giani, Aurélien Degrave, Carlos Funez Guerra, Susana Lopez, Ivan Mesonero

**Affiliations:** 1 Department of Electrical Energy Storage, Fraunhofer Institute for Solar Energy Systems, Freiburg im Breisgau, Germany; 2 Department of Sustainable Systems Engineering (INATECH), University of Freiburg, Freiburg im Breisgau, Germany; 3 Giroa-Veolia, Zamudio, Spain; 4 Azienda Elettrica Di Massagno, Massagno, Switzerland; 5 i.LECO NV, Machelen, Belgium; 6 Iberdrola, Madrid, Spain; 7 Tekniker, Gipuzkoa, Spain

**Keywords:** Energy Community, Sector Coupling, System of Systems, Demonstration Scenario, Energy Transition, Funnel Approach, Reverse Funnel Approach, Flexibility

## Abstract

**Background:**

Energy communities facilitate several advantages, including energy autonomy, reduced greenhouse gas emissions, poverty mitigation, and regional economic development. They also empower citizens with decision-making and co-ownership prospects in community renewable projects. Integrating renewable energy sources and sector coupling is a crucial strategy for flexible energy systems. However, demonstrating clean energy transition scenarios in these communities presents challenges, including technology integration, flexibility activation, load reduction, grid resilience, and business case development.

**Methods:**

Based on the system of systems approach, this paper introduces a 4-step funnel approach and a 4-step reverse funnel approach to systematically specify and detail demonstration scenarios for energy community projects. The funnel approach involves four steps. First, it selects demonstration scenarios promoting energy-efficient state-of-the-art renewable technologies and storage systems, flexibility through demand side management techniques, reduced grid dependence, and economic viability. Second, it lists all existing and planned project technologies, analysing energy flows. Third, it plans actions at different levels to implement the demonstration scenarios. Fourth, it validates the strategies using key performance indicators (KPI) to quantify the effectiveness of the planned measures. Furthermore, the reverse funnel approach delves deeper into the demonstration scenarios. The four steps involve identifying stakeholder perspectives, describing scenario scopes, listing conditions for realisation, and outlining business models, including value chains and economic assumptions.

**Results:**

This approach provides a detailed analysis of the demonstration scenarios, considering actors, objectives, boundary conditions, and business assumptions. The methodologies are exemplified in three diverse European energy communities extending across residential, commercial, tertiary, and industrial establishments, allowing power-to-x and sector coupling opportunities. The paper also suggested thirteen KPIs for validating renewable-focused energy community projects.

**Conclusions:**

Finally, the paper recommends increased collaboration between energy communities, knowledge sharing, stakeholder engagement, transparent data collection and analysis, continuous feedback, and method improvement to mitigate policy, technology, business, and market uncertainties.

## Introduction

The European Union (EU) presented the 'Fit for 55' package to reduce greenhouse gas (GHG) emissions by a minimum of 55% by 2030, boosting the EU's renewable energy target to 40% by 2030 and emphasising the sector coupling of transport, buildings, and industries ^1^. Achieving this ambitious energy transition target depends on the growing integration of renewable-based and sector-coupled energy systems owned by prosumer-driven energy communities (EC) ^2^. The EU highlighted the role of citizen energy communities (CEC) and renewable energy communities (REC) through innovative and proactive consumer participation activities ^3, 4^. These energy communities are collectives formed by citizens to produce, manage, and consume their own energy from locally installed renewable assets ^5^. ECs offer advantages such as promoting energy independence, reducing GHG emissions, combating fuel poverty, and stimulating the local economy. Additionally, they empower citizens by providing democratic authority over energy investments, allowing them to become co-owners of renewable installations and participate in the decision-making processes ^6^. ECs are backed by the Renewable Energy Directive, also termed RED II, which encourages active consumer participation in all markets and urges member countries to adopt support schemes ^7^.

Sector coupling offers innovative solutions to the flexibility requirements of energy systems by creating cost- and fuel-efficient strategies via smart integration ^8^. It enables cost-efficient investment decisions, boosts energy infrastructure use, and facilitates new storage opportunities for increased integration of renewable energy sources (RES) ^8, 9^. By converting power-to-X (P2X) and creating a system of systems (SoS), sector coupling paves the way for in-depth energy system decarbonization ^10^. The landscape of P2X technologies, which includes power-to-heat (P2H), power-to-gas (P2G), and power-to-vehicle (P2V), is an evolving one to connect diverse energy sectors and thereby solving the intermittent issue of renewables ^8^. The SoS approach connects the ECs, the utility-scale systems, the renewable energy sources (RES), the grid-level systems, and the energy market on a common platform for planning, controlling and optimisation. In the SoS approach, electricity is considered the leading energy carrier, and the power grid becomes central to decarbonising the whole energy system through maximum P2X usage, enabling cross-vector integration of energy resources and improved grid operations management. The SoS approach entails (i) energy optimisation, (ii) decarbonisation, (iii) energy efficiency, (iv) system flexibility and resilience, (v) cost- and fuel-efficient systems, and (iv) infrastructure optimisation ^11^. This holistic integration allows for addressing not only technical issues with renewable energy usage but also challenges in energy security, economics, and environmental impacts ^11^.

Integrating RES-based systems and employing sector coupling amplifies efficient and flexible intra- and inter-community energy exchange, promoting decarbonisation and optimising grid operations, and establishing a community federation controllable through a cloud-based platform ^12, 13^. The SoS approach enhances grid resilience, reduces costs, minimises distribution losses, and facilitates the management of peak loads, benefiting energy communities and grid operators ^11^. However, demonstrating clean energy transition scenarios in sector-coupled and renewable-based energy communities presents several challenges. These include formulating appropriate methods that balance multiple motivations, such as integrating diverse RES-relevant technologies effectively, activating flexibility in energy communities, decreasing load, reducing grid dependability, developing viable business cases, and establishing clear validation metrics. Diverse RES technology integration involves complexities such as technology interoperability and effective integration frameworks. For example, Lund *et al.* (2015) explored hybrid energy systems and the integration of diverse RES technologies in the context of Denmark’s energy system ^10^. Activating flexibility in energy communities is complex due to existing electricity markets, tariffs, and regulations that often hinder effective demand response, load shifting, and energy storage solutions ^14^. Energy communities face grid dependence and load management challenges due to the lack of coordinated control and incentives for customer-owned assets ^15^. The existing literature on energy community business models remains fragmented, lacking a cohesive systematization of community arrangements ^16^. Also, the need for comprehensive validation metrics to measure the performance of smart energy systems has been emphasized by Efkarpidis *et al.* (2022) ^17^. Challenges include identifying actors, objectives, boundaries, and revenue-generating business cases ^18, 19^. Therefore, overcoming these challenges requires a mix of approaches to finalise a set of demonstration scenarios and then elaborate them for detailed characterisation.

Based on these, the authors formulate the following primary research question: How does one effectively address the complexities of demonstrating clean energy transition scenarios in sector-coupled and renewable-based energy communities?

To address the research question, a comprehensive methodology and case study focused on three distant European energy communities is provided. The method consists of two novel approaches described in the Methodology section: firstly, a SoS approach to build an optimised platform integrating multiple energy sectors, and secondly, exploring clean energy transition scenarios to unlock the potential of energy communities. The demonstration scenarios for the project are then presented, highlighting planned actions, and validation strategy. The section after compares the analysed scenarios, evaluating their stakeholders, use case deployment, sector-coupling aspects, impacted markets, a preliminary business model analysis, and challenges and de-risking techniques. Finally, the paper is concluded by discussing the findings, identifying novel insights, and outlining future challenges. The SoS approach optimizes integration across diverse energy sectors, enhancing cost-effectiveness and operational efficiency in future energy systems. Clean energy transition scenarios guide efficient energy use, accelerate renewable adoption, promote low-carbon fuels, optimize energy markets, and improve integrated infrastructure. Energy communities empower local renewable energy generation, promote efficiency, foster community-based storage, enhance grid resilience through decentralized management, and facilitate peer-to-peer energy trading, contributing significantly to sustainability and reducing reliance on centralized systems.

## Methods

Based on the SoS strategies in 8, we employ a dual approach to define and analyse the demonstration scenarios. The first approach, the "funnel approach," is used to specify the demo scenarios. On the other hand, the second approach, the "reverse funnel approach," is used to provide a more detailed examination of the demo scenarios.

### The funnel approach

**Figure 1.**
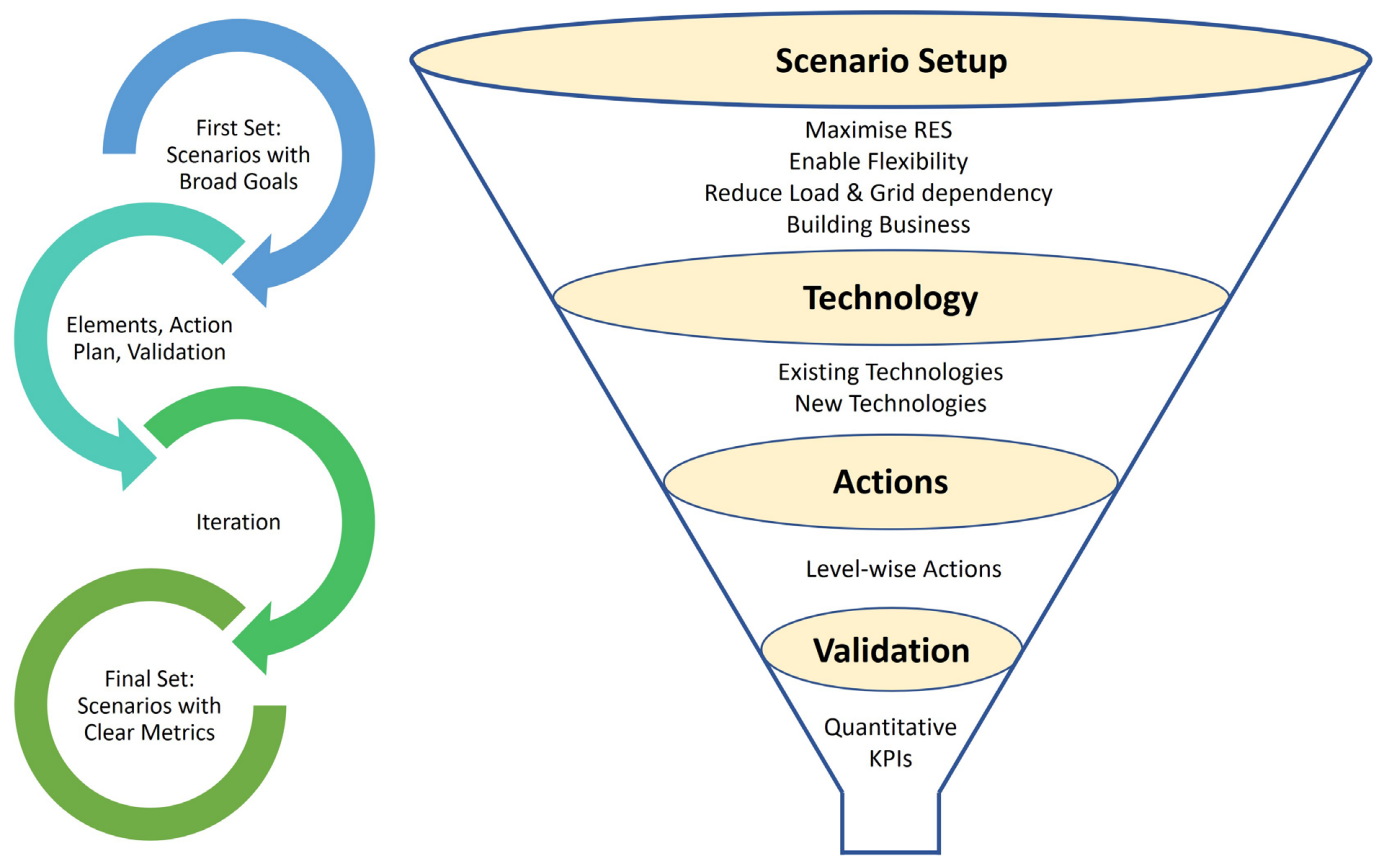
Funnel Approach to Specify the Demo Scenarios.

In the first phase, we introduce the funnel approach, illustrated in Figure 1, to specify demo scenarios in EC projects. The funnel approach provides a structured method for narrowing down and specifying demonstration scenarios. This approach is closely tied to the SoS method ^11^, which complements it by ensuring a holistic integration of the various subsystems within the EC. In energy systems, the SoS method refers to integrating and coordinating various independent subsystems, such as renewable energy sources, storage solutions, demand-side management tools, and grid infrastructure, into a cohesive and efficient overall system. Each subsystem operates autonomously, but combined with the SoS approach, they achieve broader objectives like enhancing grid reliability, increasing renewable energy penetration, reducing carbon emissions, and improving economic efficiency. While the funnel approach progressively refines and validates scenarios to meet specific project objectives, the SoS method guarantees that these scenarios are coherent and aligned with the broader goals of decarbonisation, flexibility, and economic viability.

The choice of a 4-step funnel approach is driven by the need for a structured and systematic process to refine and validate demonstration scenarios in energy projects. Each step represents a critical phase in narrowing broad concepts into actionable, feasible plans aligning with the overarching project goals. By progressing through these four steps- from scenario development, technology listing, action planning, and finally, validation, this approach ensures that each aspect of the project is thoroughly examined, integrated, and tested.

The broad objectives of such projects include decarbonising the energy system, reducing carbon footprint, enhancing grid reliability, and generating economic benefits. Beginning with defining a set of demo scenarios, we then employ the funnel approach to identify the elements, illustrate action plans, and validate them. Throughout the iterative process, we use the SoS method to ensure a holistic perspective and effective integration of various subsystems within the energy community. This combined approach aims to finalise a feasible set of demonstration scenarios with clear metrics for validation while also considering the development of a viable business case for the pilot sites. The proposed 4-step funnel approach is described below:

**1.**  At the onset of the funnel approach, first, we propose to develop a preliminary list of demonstration scenarios and constructing feasible business case models. The preliminary list of demonstration scenarios is developed using the SoS approach, prioritising scenarios that best align with the EC’s goals of maximising renewable energy integration, enhancing flexibility, reducing grid dependence, and ensuring economic viability. Each scenario is weighted based on its contribution to these objectives, providing a cohesive and effective set of scenarios for further development. The anticipated business case models underscore a platform-based approach integrating multiple stakeholders through digital platforms, enhancing scale, efficiency, and innovation. These platforms enable flexible, modular services like demand response and electrification, reducing marginal costs and fostering direct interaction between prosumers and service providers. This initial step is crucial as it allows us to focus specifically on these pivotal motivations of energy community projects and tailor the demo scenarios to promote increasing renewable energy integration, improving energy flexibility, reducing grid reliance, and fostering economic viability.
(i) To maximise the RES penetration to the existing system in ECs, we recommend integrating diverse energy-efficient state-of-the-art technologies such as P2X, district heating and cooling units (DH&C), combined heat and power plants (CHP), and e-mobility with vehicle-to-grid (V2G) charging infrastructure for bi-directional energy flows. Additionally, we aim to connect RES technologies with storage systems like batteries and pumped hydro, ensuring efficient energy management and use.
(ii) Next, we propose actively exploring and implementing opportunities for activating flexibility within the ECs through demand side management (DSM) techniques. We advocate for brainstorming and identifying strategies to effectively deploy demand response (DR) and energy efficiency measures, enabling improved load management. By actively engaging with consumers and promoting energy-conscious behaviours, we can enhance overall system flexibility and ensure the optimal use of RES.
(iii) Then, the scenarios should focus on how steps (i) and (ii) can reduce the dependability on the grid network. Reducing dependence on the grid lowers user costs by cutting electricity purchases, as seen with residential solar installations that reduce bills. It also decreases grid investment needs by alleviating strain and maintenance requirements, minimizing the need for costly upgrades.
(iv) Finally, the scenarios need to reflect the development of an energy trading platform and market for energy exchange among the EC members and the federation of ECs. This includes disseminating information at the customer level via reports, press releases and workshops to engage the community members in realising the benefits of such projects.
**2.**  In the second step, all the existing and planned technologies of the project should be listed. Focusing on sector coupling, information needs to be collected on renewable-based electricity production, centralised and decentralised heating and cooling, mobility, hydrogen production, and storage (electricity, heat, and hydrogen). Data is gathered directly from project pilots through detailed asset inventories and technical specifications, ensuring accurate information on system components and energy flows. The energy flows between different sub-pilots or locations should also be analysed in relevant cases.
**3.**  In the third step, the actions should be planned at different EC levels to implement the demonstration scenarios. At the user level, actions include collecting data from smart meters to optimize energy usage and implementing demand-side management strategies. At the EC level, integration involves developing a cloud platform for analytics and optimizing energy use within a single EC. At the federation level, actions focus on aggregating multiple communities for intercommunity energy exchange and balancing. Detailed examples of these actions can be found in the planned actions section.
**4.**  The validation of the demonstration scenarios should form the last stage in the funnel approach. A series of key performance indicators (KPIs) can quantify the effectiveness of the planned actions. The KPIs measure the energy system's performance and the energy efficiency actions, grid monitoring, and energy savings and cost savings. The results from the validation must be compared with the project's ultimate set of target metrics.

### The reverse funnel approach

**Figure 2.**
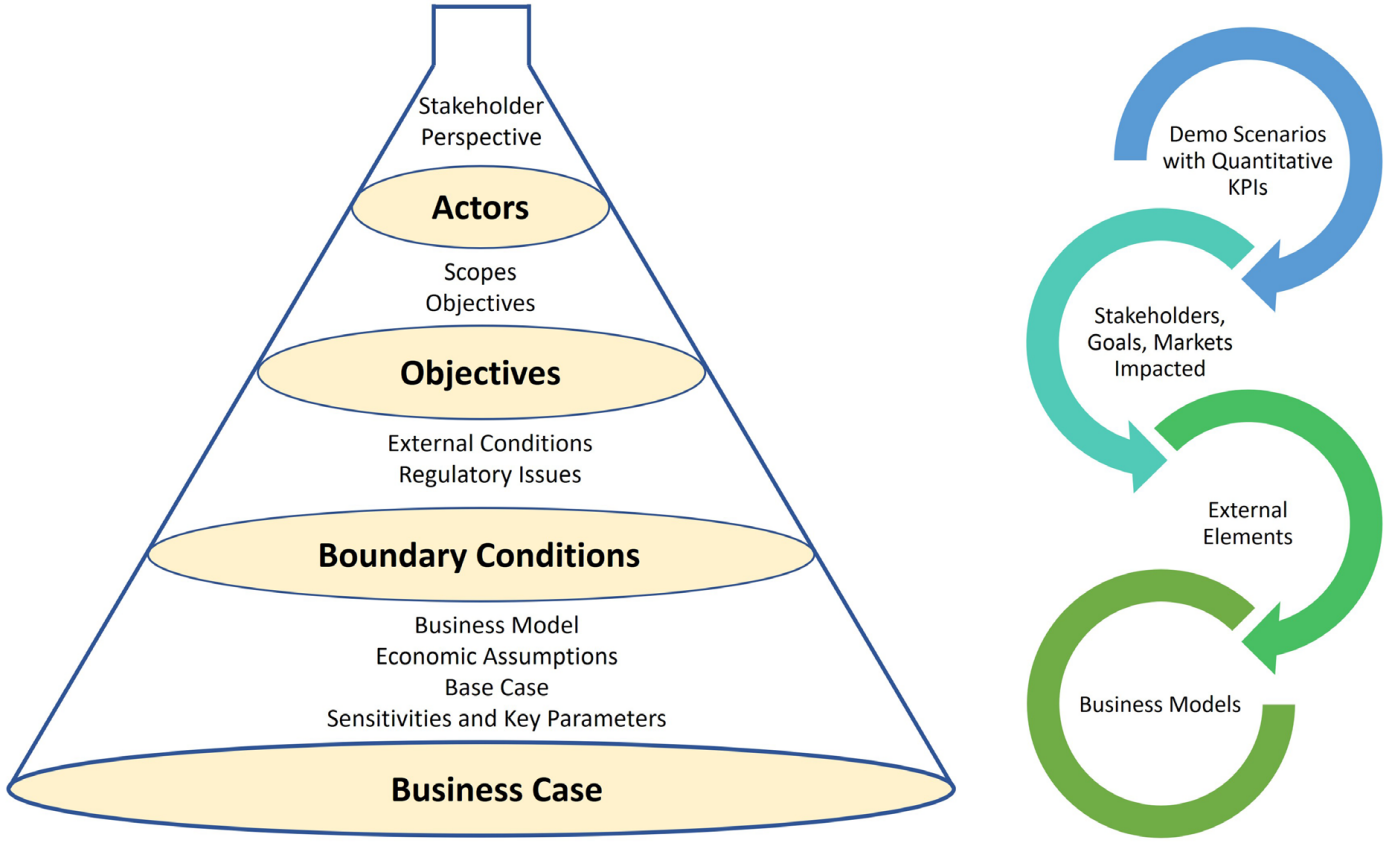
Reverse funnel approach to detail the demonstration scenarios.

After specifying the list of demo scenarios with quantitative KPIs, the demo scenarios are characterised for further analysis. A template has been prepared for structured surveys based on 11 for assessing the demo scenarios in detail. We used the reverse funnel approach, illustrated in Figure 2, to gradually widen the demonstration scenarios from actors via objectives and boundary conditions and finally indicate the preliminary business assumptions.

The 4-step reverse funnel approach is described below:

1. First, we identified the stakeholder perspective of the scenarios. For example, we categorised the actors from four points of view: (i) System, (ii) TSO, (iii) DSO, and (iv) Investor/Owner. We also identify the proposer/supporter for the scenarios.
2. Second, the scopes of the demonstration scenarios are described, detailing what is in scope and what is out of scope. The location for the scenario deployment, the rationales/key drivers, the sectors to be coupled, the technologies deployed, and the impacted markets are presented. The main rationales listed are decarbonisation, energy optimisation, infrastructure optimisation, and flexibility provision.
3. Third, the main conditions to be in place for realising the demonstration scenarios, commenting on their reasons, expectations, impact, actors/decision makers behind such conditions are listed and described. This step elaborates on ways to de-risk or secure such conditions. In addition, the main regulatory issues impacting and conditioning the feasibility of the demo scenarios are discussed.
4. Finally, the business model presents a short but comprehensive description of value chain flow: who invests, who sells/earns the product/service, who purchases/pays it, if there are pass-throughs or pass-over of costs to other stakeholders, if tariffs (or other regulated economic flows) are involved. The main implicit and explicit assumptions on economic values to be used for assessing feasibility of the scenarios are listed, including the list of externalities (positive or negative); include CO2 role and values. The best alternative(s) to reach the same goal, traditional or other competing innovative solutions, to be used as reference for comparing the economics of the scenarios against such base case are described and quantified. Furthermore, the few main parameters affecting the feasibility of the demonstration scenarios, the need of subsidies; the parameters on which future evolution is expected, are listed, and described.

## Specification of demonstration scenarios

To specify the clean energy transition scenarios in sector-coupled and renewable based energy communities, we selected the FEDECOM project pilots: (i) Spanish Virtual Green H2 Federation, (ii) Swiss Residential Hydropower Federation and (iii) BE-NL cross-country e-Mobility Federation. The pilot sites are selected at different geographical locations, with a variety of energy systems and requirements, and stretching across residential, commercial, tertiary, and industrial establishments. This allows the use of P2X technologies and enhances sector coupling opportunities. Table 3.1 presents an overview of the demonstration pilots.

**Table 3.1.** Overview of the pilots.

Pilot name	Country	Federation member	Consumer Type
Virtual Green H2 Federation	Spain (ES)	Ur Beroa Community and Bilbao Townhall	Residential, Tertiary
		Puertollano Green Hydrogen Plant	Industrial
		TMB Barcelona Station	Mobility
Residential Hydropower Federation	Switzerland (CH)	Lugaggia Innovation Community	Residential, Tertiary
		Arena Innovation Community	Residential, Tertiary
		Garamè District	Residential
Cross-country E-Mobility Federation	Belgium and Netherlands (BE-NL)	Brussels Brico Retail Community (BE)	Commercial, Residential
		Voorhout Village (NL)	Residential
		Besix HQ & Eemnes Community (NL)	Residential, Tertiary

### List of demonstration scenarios

Based on the first two stages of our funnel methodology, we finalised a list of demonstration scenarios, listed in Table 3.2. We classified sets of four sub-scenarios for each of the three selected pilot sites. The sub-scenarios are founded on the energy systems existing at the sites and explore opportunities to fulfil the project goals.

**Table 3.2.** List of demonstration scenarios.

Pilot	Scenario	Sub-scenario	Name
Virtual Green H2 Federation	S1	S1a	Integrate renewable energy generation with P2X technology
		S1b	Optimise integrated operation of DH&C systems
		S1c	Aggregate and unlock flexibility resources across federated communities
		S1d	Validate advanced control strategies under effective business models
Residential Hydropower Federation	S2	S2a	Increase the overall system efficiency through user awareness mechanisms, thus leading to a reduction in energy consumption (both thermal and electrical) and to a maximisation of local RES production
		S2b	Increase hosting capacity by enabling cross-vector demand-side flexibility to minimise power congestions and reduce grid investments
		S2c	Engage members of the energy community in an optimal energy consumption mechanism at three levels, from the individual user to the federated level
		S2d	Integrate vertical flexibility towards the balancing service providers in the form of power service by leveraging the assets installed in the federation
Cross-country e-Mobility Federation	S3	S3a	Maximise exploitation of local RES generation across energy sectors
		S3b	Unlock demand flexibility by integrating with EV charging infrastructures
		S3c	Enable sharing of locally produced energy across community members
		S3d	Demonstrate cross-country interaction of federated communities

### Technologies

In this section, Table 3.3, Table 3.4, and Table 3.5 provide the list of technologies available at the three pilots sites.

**Table 3.3.** Pilot 1 – Virtual Green H2 Federation (ES).

Sub-pilot/Building		Centralised Heating/ Cooling	Decentralised Heating/ Cooling	Electricity Production	Public Mobility	Private Mobility	Hydrogen Production	Heat Storage	Electricity Storage	Hydrogen Storage	Coupling Heating/ Cooling - Electricity
Bilbao Townhall	Casa Consistorial	2 gas boilers	17 HP and 168 internal units ^1^								X
	San Agustin	6 gas boilers and 2 chillers		PV		Only city council			Linked UPS ^2^		X
	Aznar	2 gas boilers ^3^	6 HP					X	Linked UPS ^3^		X
	Anexo		7 HP	PV					Linked UPS ^3^		X
Ur Beroa		District Heating, 1 condensing boiler, 2 boilers, 1 biomass boiler, 1 CHP		CHP PV			X	X			X
Fertiberia (Puertollano Green Hydrogen Plant)				PV 100 MW			20 MW electrolyser		5MW/ 20MWh	X	X
TMB Barcelona						only TMB city buses	2.5 MW electrolyser			X	X

^1^ As it is a historic building, the central heating system cannot serve all the spaces and therefore heat pumps were installed. The heat pumps serve either heating or cooling. ^2^ Same UPS for 3 buildings. They provide flexibility to these buildings (San Agustin, Anexo and Casa consistorial). They are connected with a micro-grid. ^3^ These boilers give heating services to Casa Consistorial and Anexo buildings.

**Table 3.4.** Pilot 2 – Residential Hydropower Federation (CH).

Sub-pilot	Centralised Heating/Cooling	Decentralised Heating/Cooling	Electricity Production	Public Mobility	Private Mobility	Hydro Storage	Heat Storage	Electricity Storage	Coupling hydro - electricity	Coupling Heating/Cooling - Electricity
Lugaggia Innovation Community (LIC)		14 HP 46 kW 13 Electric boilers 68 kW	PV 85 kW					District battery 50 kW / 60 kWh		X ^4^
Arena Innovation Community (AIC)	District Heating 550 kW _th_ biomass boiler	2 HP 17 kW 5 Electric boilers 29 kW	PV 30 kWp PV 22.4 kWp PV 83 kWp Q4-2023	DC EV charger 11 kW (V2G) + Honda-e 37 kWh			40 m ^3^ thermal tank (hot water)	District battery ^5^ 20 kW / 20 kWh		X ^4^
Garamè District (GD)		6 HP 14 kW 3 Electric boilers 10 kW	PV 49 kWp			Seasonal storage ^6^		District battery ^5^ 20 kW / 20 kWh (Q3 2024)	Seasonal storage ^6^	X ^4^

^4^ Limited to decentralised heating and cooling systems. ^5^ Technical specifications to be defined. ^6^ Seasonal hydro storage (technical and administrative feasibility to be defined with local balancing group).

**Table 3.5.** Pilot 3 – Cross-country E-Mobility Federation (BE-NL).

Sub-pilot	Centralised Heating/Cooling	Decentralised Heating/Cooling	Electricity Production	Public Mobility	Private Mobility	Heat Storage	Electricity Storage	Coupling Heating/ Cooling - Electricity
Brico Community (production & consumption) & Brico employees (consumption)			PV		DC V2G			
Voorhout Community		HP 7.25 kW in each house	PV 8 kWp in each house	1 fast charger 180 kW (new)			Community battery 50 kW / 50 kWh	X
Eemnes Community		X	PV 86.2 kWp				Community battery	
Besix HQ		HVAC system 20 kWe	PV 80 KWp		40 EV V1G 11 kW		80kW/100 kWh	X

### Planned actions

After finalising the demonstration scenarios, the next step involves deciding on the actions to be taken. The planned actions are focused at three different interaction levels: User, EC (single community), and Federation (multiple communities). The list of planned actions consists of a wide range of tasks ranging from data collection (e.g., from smart metres) and analysis, integration of energy technologies into a common platform, application of DSM strategies, to development of a business model and a trading platform, optimisation of the dispatch model. Table 3.6 shows the list of planned actions aimed at each of the pilots.

**Table 3.6.** List of planned actions at three interaction levels.

Interaction level	Planned Actions
User	● Identification of user level stakeholders (e.g., consumer, prosumer) ● Measurement and data collection including user feedback ● Data analytics, forecasting, optimisation, monitoring, and control ● Implement DSM to maximise flexibility (e.g., P2H)
EC	● Current system evaluation and future system planning ● Identification of technical and functional requirements of the cloud platform ● Architecture development of cloud platform to facilitate analytical, modelling and optimization services ● Advanced analytics for optimal control at EC level ● Apply DSM strategies at community level (e.g., district batteries, central HVAC) ● Enable peer-to-peer intra-and inter-community trading to maximise cost benefits
Federation	● Virtual aggregation of federated communities focusing on flexibility, energy exchange, and balancing services ● Apply DSM strategies at federation level (e.g., HV/MV transformer) ● Business model development for enabling inter-community energy trading

### Validation strategy

We propose the IPMVP method for validating the demonstration scenarios ^20^. Therefore, the following list of key performance indicators (KPI) are suggested.

1) *Energy Savings –* Energy savings is an indicator of the energy usage reduction after the integration of RES at the demo sites. The FEDECOM project has a set target of achieving a minimum of 20% in energy savings after optimisation has been carried out. Equation (1) has been formulated to calculate energy savings for the project ^21^.

$$E_{savings}=\frac{(E_{baseline}-E_{reporting})\pm Adjustments}{E_{baseline,adjusted}}\times 100\%\;(1)$$

where, *E _baseline_* and *E _reporting_* are the energy demands in baseline and reporting periods, respectively, and *Adjustments* are a restatement of the energy demand of the baseline and reporting periods under a common set of conditions.

2) *Cost Savings –* Cost savings or energy cost reduction translates the amount of energy savings into economic terms ^21^.

$$E_{savings,cost}=\{(E_{baseline}-E_{reporting})\pm Adjustments\}\times E_{price}\;(2)$$

where, *E _price_* is the energy price per unit at the local market.

3) *Asset Revenue –* Asset revenue accounts for the revenue from trading between the EC and the grid at the EC level.

$$\;E_{export}=E_{RES,prod}-E_{RES,cons}-E_{stored}\;(3)$$

$$\;Revenue=E_{export}\times Tariff\;(4)$$

where, *E _RES,prod_* and *E _RES,cons_* are amounts of locally produced and consume energy, *E _stored_* is the amount of locally generated energy stored in batteries, *E _export_* is the amount of locally generated energy that is exported, and *Tariff* is the unit price set by the market.

4) *Self-sufficiency –* Self-sufficiency quantifies how much of the total energy consumption is met by the local RES and is a measure of the level of grid independence ^22^.

$$\;SS_{RES}=\frac{E_{RES,cons}}{Demand_{total}}\times 100\%\;(5)$$

where, *Demand _total_* represents the total energy demand.

5) *Self-consumption –* Self-consumption indicates how much of the generated energy from the local RES is consumed at the site ^22^.

$$\;SC_{RES}=\frac{E_{RES,cons}}{E_{RES,prod}}\times 100\%\;(6)$$

6) *Avoided CO2 Emissions –* FEDECOM targets a reduction of at least 55% in GHG emissions. The calculation is based on the energy savings during a set observed period and compared with the CO2 emissions for the baseline period ^23^.

$$\begin{array}{l} CO2\;reduction=(Demand_{grid,old}-Demand_{grid,new})\times EF_{grid}+\\ \;(Demand_{fuel,old}-Demand_{fuel,new})\times EF_{fuel} \end{array}\;(7)$$

where, *Demand _grid_* [kWh] is the electricity demand from the grid during baseline period ( *Demand _grid,old_*) and observed period ( *Demand _grid,new_*), *Demand _fuel_* [kg] is the fuel demand for heating during baseline period ( *Demand _fuel,old_*) and observed period ( *Demand _fuel,new_*), *EF _grid_* [kgCO2/kWh] is the emission factor of the local electricity mix, and *EF _fuel_* [kgCO2/kg] is the emission factor of fuel consumed for heating.

7) *Peak Shaving –* Peak shaving determines a decrease in the maximum electrical or thermal load compared between the baseline and reporting periods ^23^.

$$\;Peak\;Shaving=\frac{P_{old,max}-P_{new,max}}{P_{old,max}}\times 100\%\;(8)$$

where, *P _old,max_* [kW] and *P _new,max_* [kW] are the maximum load demands in baseline and reporting periods, respectively.

8) *Predictability of PV Production and Electricity Demand –* These measure the accuracy level in forecasting the level of PV production from the locally installed system and the electrical load demand.

$$\;PA_{x}=\frac{E_{forecasted}}{E_{realtime}}\times 100\%\;(9)$$

where, *E _forecasted_* [kWh] and *E _realtime_* [kWh] are estimated and actual levels of PV production/load demand in the observed period, respectively.

9) *Grid Operating Margin –* Grid operation margin expresses the profitability of a grid operator by considering the electricity sales revenue and the grid operating expenses.

$$\;Grid_{op,margin}=\frac{Operating\;expenses}{Revenue}\;(10)$$

where, *Operating expenses* [€] are costs incurred in running the grid and *Revenue* [€] is earned from the sale of electricity.

10) *Grid Forecasting Offset –* Grid forecasting offset measures the accuracy of the forecasting system in place at a grid operator. The normalised Mean Absolute Error (NMAE) can be used as the metric for the grid forecasting offset.

$$\;NMAE=\frac{1}{n}\times \sum \frac{\left|Forecast_{i}-Actual_{i}\right|}{Mean_{actual}}\;(11)$$

where, *n* is the number of forecasts, *Forecast _i_* is the forecasted value of the voltage for time i, *Actual _i_* is the actual value of the voltage for time i, and *Mean _actual_* is the mean of the actual values.

11) *Grid State Estimation –* Grid state estimation is essential in obtaining the electricity grid's real-time state. For this measurement, a Skill Score to compare the performance of the proposed method to a 'zero hold' method can be used.

$$\;Skill\;Score=1-\frac{RMSE_{proposed}}{RMSE_{hold}}\;(12)$$

where, *RMSE _proposed_* and *RMSE _hold_* are the Root Mean Squared Errors of the proposed, and the ‘zero hold’ method, respectively.

12) *Grid Electricity Usage Reduction –* Grid electricity usage reduction measures the reduced electricity drawn from the grid.

$$\;E_{grid,reduced}=Demand_{grid,old}-Demand_{grid,new}\;(13)$$

where, *Demand _grid_* [kWh] is the electricity demand from the grid during baseline period ( *Demand _grid,old_*) and observed period ( *Demand _grid,new_*).

13) *Grid CAPEX and OPEX Reduction –* Grid CAPEX and OPEX reduction quantifies the savings after the implementation of the project.

$$CAPEX_{grid,reduced}=\frac{CAPEX_{grid,old}-CAPEX_{grid,new}}{CAPEX_{grid,old}}\times 100\%\;(14)$$

$$OPEX_{grid,reduced}=\frac{OPEX_{grid,old}-OPEX_{grid,new}}{OPEX_{grid,old}}\times 100\%\;(15)$$

where, *CAPEX _grid_* [€] is the capital expenditures during baseline period ( *CAPEX _grid,old_*) and observed period ( *CAPEX _grid,new_*) and *OPEX _grid_* [€] is the operating expenses during baseline period ( *OPEX _grid,old_*) and observed period ( *OPEX _grid,new_*).

## Detailing the demonstration scenarios

### Actors/stakeholders

For the FEDECOM project, the identified actors or stakeholders are classified as shown in Table 4.1.

**Table 4.1.** List of actors/stakeholders.

Pilot	Entity	System/ Technical Enabler	TSO	DSO	Investor/ Owner	Energy Network	Energy Supply Chain
1	Ur Beroa Community and Bilbao Townhall	-	-	-	Yes	-	Yes
	Puertollano Green Hydrogen Plant	Yes	-	-	Yes	Yes	Yes
	TMB Barcelona Station	Yes	-	-	Yes	Yes	Yes
2	Residential Hydropower Federation	-	-	Yes	Yes	Yes	-
3	Cross-country E-Mobility Federation	Yes	-	-	-	-	-

### Sector coupling

Each of the sub-pilots’ experience interaction of a variety of energy resources. The sectors coupled are presented in Table 4.2

**Table 4.2.** Overview of the sectors coupled at the pilot sites.

Pilot	Technologies	Main Focus
Ur Beroa Community	PV production, H2 generation	Renewable energy integration, Substituting gas with H2
Bilbao Townhall	Gas and electric vectors	Optimising thermal management
Puertollano Green Hydrogen Plant	Industrial sector, Green hydrogen	Hydrogen supply to the industrial sector
TMB Barcelona Station	Mobility sector, Hydrogen supply	Hydrogen supply for mobility and industrial sectors
Residential Hydropower Federation	Thermal, electricity, EV sectors	Integration across multiple sectors
Cross-country E-Mobility Federation	Thermal, electricity, EV sectors	Integration across multiple sectors

### Impacted markets

Table 4.3 overviews different impacted markets for the FEDECOM pilot sites. These markets are impacted because the ECs at each pilot site interacts with various market mechanisms through its load and installed generation capacity, affecting energy trading, balancing, and flexibility services. However, the degree of impact can vary: for instance, smaller pilots (in terms of comparatively smaller generation capacities) may have limited influence on broader markets compared to large-scale projects. Regulatory issues include ensuring compliance with market rules and participating in market platforms. Regulatory frameworks must support integrating these pilot projects into existing markets, addressing aspects such as access, data sharing, and the fair treatment of different market participants.

**Table 4.3.** Overview of the impacted markets at the pilot sites.

Pilot	Medium Voltage	Low Voltage Day Ahead Energy Market	Day Ahead Balancing Market	Intraday Energy Market	Intraday Balancing Market	Flexibility Market	Capacity Market	Ancillary Services Market
Ur Beroa Community and Bilbao Townhall	Yes	Yes	Yes	Yes	Yes	Yes	-	-
Puertollano Green Hydrogen Plant	Yes	Yes	Yes	Yes	Yes	Yes	-	-
TMB Barcelona Station	Yes	Yes	Yes	Yes	Yes	Yes	-	-
Residential Hydropower Federation	Yes	Yes	Yes	Yes	Yes	Yes	-	-
Cross-country E-Mobility Federation	-	Yes	Yes	Yes	Yes	Yes	Yes	Yes

### Business model analysis

***Pilot 1: Virtual Green H2 Federation (ES).*** Ur Beroa Community and Bilbao Townhall are managed by the R&D department of GIROA S.A. (member of VEOLIA Group), which is an energy and environment management services company The aims and objectives of the FEDECOM project match GIROA’s Energy Service Companies (ESCO) contracts and Energy Performance Contracting (EPC) commitments to implement RES, optimise energy efficiency and advanced systems management. Scenarios S1a and S1b align with GIROA’s EPC obligations of helping its customers shift to RES, and enable energy and cost savings, respectively. As per the demo scenarios, GIROA shares energy and cost savings with its customers, which incentivizes DH&C optimization strategies. The FEDECOM project offers extra value and an edge over its competitors for analysing, tracking and operating installations. The company also aims to transition the installations towards predictive control systems.

The list of manufacturers of the small electrolysers required for Ur Beroa is small and the issue is compounded by high demands and long delivery times due to present supply chain challenges. The operations of upgrading the installations at Bilbao Townhall is restricted by the active use of the buildings but this can be bypassed by working outside the active office hours. Pilots P1b and P1c are both impacted by external conditions of hydrogen demand from the fertiliser company and the captive bus fleet, the fertiliser company consumption profile, the bus refuelling window, and the electricity price.

The business model for Ur Beroa encompasses a reduction in gas consumption by the CHP unit and thus resulting in cost savings. During low heating demand periods, the PV generated electricity can feed into the local energy market. The optimised thermal service at Bilbao also results in energy and cost savings, raising the economic efficiency of the contracts. The business model for Puertollano is made up of the Iberdrola company and the Fertiberia company. At TMB Barcelona Station, business is between Iberdrola and TMB. The Iberdrola company is in the charge of the hydrogen production facility and the hydrogen refuelling stations at P1b and P1c, respectively. Fertiberia runs the fertiliser plant and purchases hydrogen from Iberdrola. Similarly, Iberdrola supplies hydrogen to TMB for its refuelling stations.

***Pilot 2: Residential Hydropower Federation (CH).*** Azienda Elettrica di Massagno (AEM), along with other partners in the FEDECOM consortium, supports and manages the ECs in P2. Present conditions at the pilot sites are conducive for demo scenarios S2a and S2b. Smart metres are already installed at the end-user level. A communication infrastructure for data collection is in place. At the user level, there is interest in using the FEDECOM tools and also interest in being involved in DSM techniques.

There are a few regulatory issues affecting P2. Swiss law does not permit the creation of virtual energy communities within the national borders and allows only one point of delivery between the energy community and the DSO ^24^. Additionally, the law does not permit energy trading between different energy communities. The setting up of an energy community entails the use of a local and private distribution network for energy trading.

The business case for the Swiss Case is categorically defined for the demo scenarios. In S2a, the asset owner or final user has a leading role in the business model. The user will be informed about the performance metrics and improvement aspects of its assets. This information will be disseminated by the energy community owner through the FEDECOM tools/services. The user is then responsible for further actions in order to improve the efficiency of its assets. Through the implementation of DSM techniques within S2b, the local flexibility will be utilised to improve the hosting capacity of the distribution infrastructure. Consequently, this will play a key role in mitigating congestion and overvoltage events, as well as reducing the necessity for grid investments. The model for S2c encourages local energy consumption and energy exchange at the intra-community level first, and then at the inter-community level, with a final and most expensive option being energy purchase from external retailers. In S2d, the local flexibility available to each user, is aggregated at the federal level to be offered to the local DSO or on the local balancing market. This service is intended to facilitate the participation of small renewable assets, such as heat pumps or batteries, in these markets. The remuneration obtained from these services will facilitate the amortisation of the initial investment made by each asset owner.

***Pilot 3: Cross-country E-Mobility Federation (BE-NL).*** The establishment of S3a is dependent upon the funding scheme as the PV infrastructure and the EV charging network is being privately funded by Brico and additional funding may be required. There is also a need to draw up favourable government regulations and policies for this pilot. The S3b scenario requires active participation and engagement from the consumers for adoption of the RES technologies. Successful implementation of the demo scenarios calls for integrated action from all the relevant stakeholders with plans covering goals, timelines, milestones, risk management, contingencies, and funding.

A regulatory issue impacting P3 consists of requiring necessary permits and approvals from local authorities and regulatory bodies for installing the RES infrastructure. Setting up connections between the different systems and between the local energy communities and the grid is challenging and can necessitate approval from grid operators and regulatory bodies. Furthermore, regulations regarding net metering and feed-in tariffs, energy storage and demand response, privacy and data protection, and safety and liability will need to be considered and complied with.

### Challenges

The pilots are bounded by a set of external conditions and their implementation at the pilots brings forth a set of challenges, unique and specific to each of the scenarios. These external conditions and challenges are identified from the survey responses of the pilot leaders.

Bottlenecks affecting scenarios 1a, 1b, and 1c at the Ur Beroa Community are the limited number of manufacturers for small electrolysers that meet the requirements of the planned installations and the consequent long delivery times. These can delay the installation of the proton exchange membrane (PEM) electrolyser at the site; however, the risks can be reduced by contacting the manufacturer of choice early to guarantee delivery according to the planned project timeline. The buildings of the Bilbao Townhall are actively in use, resulting in limitations in upgrading the installations and hampering the comfort level of the users. There are also constraints in budget and space. Testing and upgrading of the tasks can be done outside office hours. Scenario 1d at the Ur Beroa Community and Bilbao Townhall is contingent upon the regulatory issues of installing the hydrogen supply pipe with the thermal management of the community dependent on gas prices. Scenarios for the Puertollano Plant are dependent upon the hydrogen demand from the fertiliser company, the consumption profile, and the electricity price. Scenario 1c requires optimised operation of the battery storage system with the large-scale solar production unit to meet the plant’s electricity demand. De-risking can be done by having a fixed demand and consumption profile from the fertiliser company while for scenario 1d, de-risking can be achieved by establishing a Power Purchase Agreement (PPA) at a competitive price between the Puertollano Plant and the local fertiliser company. Scenarios for the TMB Barcelona Station are dependent upon the hydrogen demand from the fleet of buses, the refuelling window, and the electricity price. These external conditions can be managed by having a fixed demand and fixed fuelling window for the bus fleet. The incorporation of the hydrogen storage tanks into the refuelling station can mitigate the challenges of scenario 1c. Additionally, de-risking for scenario 1d calls for setting up PPAs at competitive prices: one between Iberdrola and the DSO for buying electricity for the hydrogen production system and one between Iberdrola and the TMB Barcelona Station for the selling of hydrogen for the bus fleet.

The external conditions at pilots 2 and 3 are categorised based on the sub-scenarios. Scenario 2a is dependent on participation of a large number of end-users, installation of smart metres and a communication infrastructure between the metres and the FEDECOM platform. Successful operation of scenario 2b calls for setting up of devices that are suitable for demand side management and also requires user willingness to participate. Scenario 2c requires suitable regulations for the energy exchange market and attractive prices to promote exchange. Updating the market rules for ancillary services and providing incentives for communities, with surplus energy production, to increase participation in the ancillary services market is key for scenario 2d. The de-risking strategies here involve making the digital tools readily available, leveraging smart metres and existing communication infrastructures to gather data and enable efficient energy management, introducing incentive mechanisms, and urging users in actively shaping their energy consumption. Additionally, attractive pricing structures, complemented by price caps and suitable regulations, are to be implemented to stimulate energy exchange within local and federation markets. Market rules are to be regularly updated to incentivize participation in ancillary services markets, while surplus energy production is supported through dedicated mechanisms. These collective efforts aim to enhance energy efficiency, reduce consumption, and foster a sustainable residential energy ecosystem.

Scenario 3a is subject to private funding currently, and additional funding may be called for in the future. There is also a need for government policies and regulations that are favourable to the pilot. Scenario 3b requires active participation and engagement from the consumers, energy storage to ensure stable and reliable performance, and advanced energy management systems for managing and optimising the flow of energy. Implementation of scenario 3c is dependent on adequate grid and IT infrastructure to facilitate the transfer of energy and support the communication and data transfer between the various actors, respectively. Scenario 3d is contingent upon regulatory frameworks permitting inter-community energy trading, ensuring interconnections between communities, and setting up a tariff structure for energy exchange. To secure these conditions, a comprehensive plan and strategy should be developed, involving all relevant stakeholders and actors, including government, industry, and consumers. This plan should include clear goals, timelines, and milestones, as well as risk management and contingency plans to address any potential challenges or obstacles. In addition, collaboration and partnerships between various actors and stakeholders can help to secure funding and resources, as well as promote the adoption and implementation of the UC.

The implementation of clean energy scenarios in the pilots brings with it diverse challenges such as availability of installation units, regulatory hurdles, existing market rules, and user interest to participate. Mitigating these challenges involve strategies of early planning and engagement with suppliers and drawing up PPAs for favourable terms and prices. In addition, utilising digital tools, smart meters, and existing grid infrastructure, while encouraging user participation through incentive mechanisms and feed-in tariffs, play a pivotal role in a successful operation of energy communities. Advancing the clean energy transition requires for a multi-sectoral approach in planning, collaboration and building up regulatory frameworks.

## Conclusions

### Novelties

The paper provided two approaches to define, analyse, and validate demonstration scenarios for energy community projects systematically and comprehensively. The novelties are listed below:

1. The paper presents a 4-step funnel approach that can be used to specify demonstration scenarios in energy community projects, focusing on maximising renewable energy integration, enhancing flexibility, reducing grid reliance, and fostering economic viability.
2. The paper presents a 4-step reverse funnel approach that can be used to detail the demonstration scenarios, considering stakeholder perspectives, scopes, conditions, and business models.
3. The paper exemplified the approaches for three pilots of multiple energy communities.
4. The paper also suggested thirteen KPIs for validating renewable-focused energy community projects.

### Limitations and recommendations

We identified four limitations for the described approaches. First, the approaches simplified the complex real-world energy community projects. The interaction between distant communities to make a federation is crucial and should be considered as realistically as possible. Second, although we have specified and detailed the demonstration scenarios in a general manner, it is possible that the application of the approaches is not universal, especially in the case of communities with different characteristics. Third, the methodology relies heavily on data collection and analysis, which is a big challenge, especially in energy communities where multiple stakeholders are engaged. Finally, policy changes, technology uncertainties, business model sustainability, and market dynamics may heavily impact the methodology.

Therefore, we recommend four steps for a better demonstration. First, increase collaboration between energy communities and encourage knowledge sharing of different projects. The engagement of all stakeholders is an absolute necessity. Second, applying these approaches to energy community projects with diverse characteristics will lead to broader adoption. Third, the data collection and analysis process should be transparent and robust. Finally, continuous feedback and improvement of the method could decrease policy, technology, business, and market uncertainties.

In summary, the paper proposes a novel and comprehensive methodology with case studies focused on three distant European energy communities. However, several limitations are also identified, which should be considered for continuous improvement.

## Data Availability

Zenodo: Template and Results of Demo Scenarios Analysis Survey https://doi.org/10.5281/zenodo.10039532 ^25^ This project contains the following underlying data: Survey_Results_Pilots_1_2_3.pdf (Results of the Demo Scenario Analysis for each of the FEDECOM project pilot sites) Demo Scenario Analysis Main Template.pdf Data are available under the terms of the Creative Commons Attribution 4.0 International license (CC-BY 4.0).
