# Supplementary material for: Demonstrating clean energy transition scenarios in sector-coupled and renewable-based energy communities
Source: Open Res Eur. 2024 Sep 2;3:193. Originally published 2023 Nov 9. [Version 2] doi: 10.12688/openreseurope.16693.2 (PMC11443228; doi:10.12688/openreseurope.16693.2)
Supplement: Peer review history

# Reviewer report: Reviewer response for version 2

**DOI:** 10.21956/openreseurope.19934.r44251

**Published:** 2024-09-30

**Authors:** Giuseppe Aruta

**Affiliations:** 1 Università degli Studi di Napoli Federico II, Napoli, Italy

Two approaches for methodically and thoroughly creating, assessing, and evaluating demonstration scenarios in energy community projects were provided in the paper. Overall, the text offers a thorough framework, which allows the reader to understand the idea.

 Following comments from previous revisions, in my opinion, the article is ready for indexing.

Is the rationale for developing the new method (or application) clearly explained?

Yes

Is the description of the method technically sound?

Yes

Are the conclusions about the method and its performance adequately supported by the findings presented in the article?

Yes

If any results are presented, are all the source data underlying the results available to ensure full reproducibility?

Yes

Are sufficient details provided to allow replication of the method development and its use by others?

Yes

Reviewer Expertise:

Buildings, Energy Communities, Optimization, Artificial intelligence

I confirm that I have read this submission and believe that I have an appropriate level of expertise to confirm that it is of an acceptable scientific standard.

# Reviewer report: Reviewer response for version 2

**DOI:** 10.21956/openreseurope.19934.r43892

**Published:** 2024-09-13

**Authors:** Elisa Marrasso

**Affiliations:** 1 University of Sannio, Benevento, Italy

The Authors have addressed all comments. They have improved the paper. In my opinion the manuscript is ready for the indexing.

Is the rationale for developing the new method (or application) clearly explained?

Yes

Is the description of the method technically sound?

Partly

Are the conclusions about the method and its performance adequately supported by the findings presented in the article?

Yes

If any results are presented, are all the source data underlying the results available to ensure full reproducibility?

Partly

Are sufficient details provided to allow replication of the method development and its use by others?

Partly

Reviewer Expertise:

Energy Communities, energy conversion systems, positive enrgy districts

I confirm that I have read this submission and believe that I have an appropriate level of expertise to confirm that it is of an acceptable scientific standard.

# Reviewer report: Reviewer response for version 1

**DOI:** 10.21956/openreseurope.18029.r37306

**Published:** 2024-01-31

**Authors:** Joao Lagarto

**Affiliations:** 1 ISEL - Instituto Superior de Engenharia de Lisboa, Instituto Politécnico de Lisboa,, Lisboa, Portugal

The authors propose a 4-step funnel approach and a 4-step reverse funnel approach to address the complexities of demonstrating clean energy transition scenarios in sector-coupled and renewable-based energy communities. The paper is well and clearly written. Some suggestions to improve the quality of the paper:

 1. Provide more details on the referred complexities. How are these complexities dealt in the literature? Provide more references.

 2. Provide more details regarding impacted markets (table 4.3). Why these markets are impacted and how, in what degree? It seems from the load and installed generation capacity of some of these pilots have little impact on broader electricity markets. Moreover, are there any regulatory issues regarding the participation in these markets?

 3. Provide more details about sub-scenarios c and d for pilots 1 and 3, as is done for pilot 2.

 4. In the challenges subsection for pilot 2, a PPA between Puertollano plant and the fertilizer company is not also a viable solution to lower risk?

 5. Regarding KPI. Grid forecasting offset refers to the forecasting of which variable? If applied to more than one, please give examples. Grid state estimation: RMSE is referring to which variable.

 6. In the first time an abbreviation is used write for what it stands for.

Is the rationale for developing the new method (or application) clearly explained?

Partly

Is the description of the method technically sound?

Partly

Are the conclusions about the method and its performance adequately supported by the findings presented in the article?

Yes

If any results are presented, are all the source data underlying the results available to ensure full reproducibility?

Partly

Are sufficient details provided to allow replication of the method development and its use by others?

Partly

Reviewer Expertise:

Power systems economics, electricity markets

I confirm that I have read this submission and believe that I have an appropriate level of expertise to confirm that it is of an acceptable scientific standard, however I have significant reservations, as outlined above.

# Response

**Published:** 2024-08-21

**Authors:** Md Nasimul Islam Maruf

**Affiliations:** Electrical Energy Storage, Fraunhofer Institute for Solar Energy Systems, Freiburg, Baden-Württemberg, Germany

Dear Joao, Thank you very much for reviewing our paper and for providing your valuable feedback. We have taken into account your comments and have revised accordingly. We are presenting here a point-by-point response to your comments.    

*JL: ‘1. Provide more details on the referred complexities. How are these complexities dealt in the literature? Provide more references.’*

**Authors:** We have expanded on the complexities and covered them based on literature. ‘ *Diverse RES technology integration involves complexities such as technology interoperability and effective integration frameworks. For example, Lund et al. (2015) explored hybrid energy systems and the integration of diverse RES technologies in the context of Denmark’s energy system ^8^. Activating flexibility in energy communities is complex due to existing electricity markets, tariffs, and regulations that often hinder effective demand response, load shifting, and energy storage solutions ^22^. Energy communities face grid dependence and load management challenges due to the lack of coordinated control and incentives for customer-owned assets ^23^. The existing literature on energy community business models remains fragmented, lacking a cohesive systematization of community arrangements ^24^. Also, the need for comprehensive validation metrics to measure the performance of smart energy systems has been emphasized by Efkarpidis et al. (2022) ^25^.’*    

*JL: ‘2. Provide more details regarding impacted markets (table 4.3). Why these markets are impacted and how, in what degree? It seems from the load and installed generation capacity of some of these pilots have little impact on broader electricity markets. Moreover, are there any regulatory issues regarding the participation in these markets?’*

**Authors:** We have revised the section on ‘Impacted markets’ to explain how the energy communities can have impacts and on which services. The load and installed generation capacities of these pilots are varying and depending on these capacities, their degree of impact will vary. There are also regulatory issues regarding the participation of the energy communities in the markets. We have now mentioned regulatory issues in this section on ‘Impacted markets’ and have provided details on regulatory issues for different studied scenarios in the section on ‘Challenges’. *‘These markets are impacted because the ECs at each pilot site interacts with various market mechanisms through its load and installed generation capacity, affecting energy trading, balancing, and flexibility services. However, the degree of impact can vary: for instance, smaller pilots (in terms of comparatively smaller generation capacities) may have limited influence on broader markets compared to large-scale projects. Regulatory issues include ensuring compliance with market rules and participating in market platforms. Regulatory frameworks must support integrating these pilot projects into existing markets, addressing aspects such as access, data sharing, and the fair treatment of different market participants.’*    

*JL: ‘3. Provide more details about sub-scenarios c and d for pilots 1 and 3, as is done for pilot 2.’* **Authors:** We have expanded our discussion with details for the sub-scenarios for all pilots. *‘Scenario 1d at the Ur Beroa Community and Bilbao Townhall is contingent upon the regulatory issues of installing the hydrogen supply pipe with the thermal management of the community dependent on gas prices.’* *‘Scenario 1c requires optimised operation of the battery storage system with the large-scale solar production unit to meet the plant’s electricity demand.’* *‘The incorporation of the hydrogen storage tanks into the refuelling station can mitigate the challenges of scenario 1c. Additionally, de-risking for scenario 1d calls for setting up PPAs at competitive prices: one between Iberdrola and the DSO for buying electricity for the hydrogen production system and one between Iberdrola and the TMB Barcelona Station for the selling of hydrogen for the bus fleet.’* *‘Scenario 3d is contingent upon regulatory frameworks permitting inter-community energy trading, ensuring interconnections between communities, and setting up a tariff structure for energy exchange.’*    

*JL: ‘4. In the challenges subsection for pilot 2, a PPA between Puertollano plant and the fertilizer company is not also a viable solution to lower risk?’*

**Authors:** Yes, a PPA between the Puertollano plant and the local fertiliser company can be a viable solution to lower risks. We have added this into our discussion. *‘De-risking can be done by having a fixed demand and consumption profile from the fertiliser company while for scenario 1d, de-risking can be achieved by establishing a Power Purchase Agreement (PPA) at a competitive price between the Puertollano Plant and the local fertiliser company.’*    

*JL: ‘5. Regarding KPI. Grid forecasting offset refers to the forecasting of which variable? If applied to more than one, please give examples. Grid state estimation: RMSE is referring to which variable.’*

**Authors:** In both cases we referred to forecast error and state estimation of the voltages, since this is the output of the state estimation model, which will then be used to define constraints in the optimization algorithms.    

*JL: ‘6. In the first time an abbreviation is used write for what it stands for.’*

**Authors:** We have gone through the manuscript and corrected the abbreviations.

# Reviewer report: Reviewer response for version 1

**DOI:** 10.21956/openreseurope.18029.r36214

**Published:** 2023-12-15

**Authors:** Elisa Marrasso

**Affiliations:** 1 University of Sannio, Benevento, Italy

The paper presented two methods for systematically and comprehensively defining, analyzing, and validating demonstration scenarios in energy community projects. Overall, the structure is clear, and the content provides a detailed framework. However, some critical revisions and comments can enhance clarity and precision:

- The introduction can be improved given more details about the topic and providing the novelty of this work in the context of the recent scientific literature.
- Specify the relationship between the funnel approach, Figure 1, and the SoS method in a more explicit manner for better clarity.
- 4-Step Funnel Approach: Consider providing a brief rationale for choosing a 4-step funnel approach.
- Step 1: Developing Demo Scenarios. Please, specify the criteria or methodology for developing the preliminary list of demonstration scenarios. How are motivations prioritized or weighted?
- Provide a concise overview of the envisioned business case models to ensure a clear understanding.
- Step 1 (iii): Reducing Dependence on the Grid: Please, clarify how reduced dependence on the grid contributes to cost savings at the user level and lower investments in grid operation. Consider providing examples or case studies to illustrate potential cost savings.
- Step 2: Listing Existing and Planned Technologies: Specify how information on technologies will be collected and analyzed for sector coupling.
- Step 3: Planning Actions at Different EC Levels: Please, provide concrete examples of actions at each level to help readers understand the practical implications of data collection at the user level, integration at the EC level, and aggregation at the federation level.
- Clarify what "PEM electrolyser" stands for upon first mention for readers unfamiliar with the term (Proton Exchange Membrane electrolyser).
- Conclude the section “Challenges” by summarizing the key challenges and potential solutions discussed in the pilots. This will reinforce the main takeaways for the reader.

Is the rationale for developing the new method (or application) clearly explained?

Yes

Is the description of the method technically sound?

Partly

Are the conclusions about the method and its performance adequately supported by the findings presented in the article?

Yes

If any results are presented, are all the source data underlying the results available to ensure full reproducibility?

Partly

Are sufficient details provided to allow replication of the method development and its use by others?

Partly

Reviewer Expertise:

Energy Communities, energy conversion systems, positive enrgy districts

I confirm that I have read this submission and believe that I have an appropriate level of expertise to confirm that it is of an acceptable scientific standard, however I have significant reservations, as outlined above.

# Response

**Published:** 2024-08-21

**Authors:** Md Nasimul Islam Maruf

**Affiliations:** Electrical Energy Storage, Fraunhofer Institute for Solar Energy Systems, Freiburg, Baden-Württemberg, Germany

Dear Elisa, Thank you very much for reviewing our paper and for providing your valuable feedback. We have taken into account your comments and have revised accordingly. We are presenting here a point-by-point response to your comments.  

*EM: ‘The introduction can be improved given more details about the topic and providing the novelty of this work in the context of the recent scientific literature.’*

**Authors: **We have improved the introductory section to include more on energy communities and their legal support frameworks, sector coupling technologies and the System-of-Systems approach. We have expanded on the novelty of this work. *‘These energy communities are collectives formed by citizens to produce, manage, and consume their own energy from locally installed renewable assets ^20^.’* *‘ECs are backed by the Renewable Energy Directive, also termed RED II, which encourages active consumer participation in all markets and urges member countries to adopt support schemes ^21^.’* *‘The landscape of P2X technologies, which includes power-to-heat (P2H), power-to-gas (P2G), and power-to-vehicle (P2V), is an evolving one to connect diverse energy sectors and thereby solving the intermittent issue of renewables ^6^.’* *‘In the SoS approach, electricity is considered the leading energy carrier, and the power grid becomes central to decarbonizing the whole energy system through maximum P2X usage, enabling cross-vector integration of energy resources and improved grid operations management.’* *‘This holistic integration allows for addressing not only technical issues with renewable energy usage but also challenges in energy security, economics, and environmental impacts ^9^.’* *‘The method consists of two novel approaches described in the Methodology section: firstly, a SoS approach to build an optimised platform integrating multiple energy sectors, and secondly, exploring clean energy transition scenarios to unlock the potential of energy communities.’* *‘The SoS approach optimizes integration across diverse energy sectors, enhancing cost-effectiveness and operational efficiency in future energy systems. Clean energy transition scenarios guide efficient energy use, accelerate renewable adoption, promote low-carbon fuels, optimize energy markets, and improve integrated infrastructure. Energy communities empower local renewable energy generation, promote efficiency, foster community-based storage, enhance grid resilience through decentralized management, and facilitate peer-to-peer energy trading, contributing significantly to sustainability and reducing reliance on centralized systems.’*  

*EM: ‘Specify the relationship between the funnel approach, Figure 1, and the SoS method in a more explicit manner for better clarity.’*

**Authors: **We have revised the section and broadened on the relationship between the funnel approach and the SoS method. *‘The funnel approach provides a structured method for narrowing down and specifying demonstration scenarios. This approach is closely tied to the SoS method ^9^, which complements it by ensuring a holistic integration of the various subsystems within the EC. In energy systems, the SoS method refers to integrating and coordinating various independent subsystems, such as renewable energy sources, storage solutions, demand-side management tools, and grid infrastructure, into a cohesive and efficient overall system. Each subsystem operates autonomously, but combined with the SoS approach, they achieve broader objectives like enhancing grid reliability, increasing renewable energy penetration, reducing carbon emissions, and improving economic efficiency. While the funnel approach progressively refines and validates scenarios to meet specific project objectives, the SoS method guarantees that these scenarios are coherent and aligned with the broader goals of decarbonisation, flexibility, and economic viability.’*  

*EM: ‘4-Step Funnel Approach: Consider providing a brief rationale for choosing a 4-step funnel approach.’*

**Authors: **We have revised the section to include an explanation for choosing a 4-step funnel approach. *‘The choice of a 4-step funnel approach is driven by the need for a structured and systematic process to refine and validate demonstration scenarios in energy projects. Each step represents a critical phase in narrowing broad concepts into actionable, feasible plans aligning with the overarching project goals. By progressing through these four steps- from scenario development, technology listing, action planning, and finally, validation, this approach ensures that each aspect of the project is thoroughly examined, integrated, and tested.’*  

*EM: ‘Step 1: Developing Demo Scenarios. Please, specify the criteria or methodology for developing the preliminary list of demonstration scenarios. How are motivations prioritized or weighted?’*

**Authors: **We have revised the details of Step 1 and expanded on how the preliminary list of demonstration scenarios are developed. *‘The preliminary list of demonstration scenarios is developed using the SoS approach, prioritising scenarios that best align with the EC’s goals of maximising renewable energy integration, enhancing flexibility, reducing grid dependence, and ensuring economic viability. Each scenario is weighted based on its contribution to these objectives, providing a cohesive and effective set of scenarios for further development.’*  

*EM: ‘Provide a concise overview of the envisioned business case models to ensure a clear understanding.’*

**Authors: **We have expanded on the details of the business case models in Step 1. *‘The anticipated business case models underscore a platform-based approach integrating multiple stakeholders through digital platforms, enhancing scale, efficiency, and innovation. These platforms enable flexible, modular services like demand response and electrification, reducing marginal costs and fostering direct interaction between prosumers and service providers.’*  

*EM: ‘Step 1 (iii): Reducing Dependence on the Grid: Please, clarify how reduced dependence on the grid contributes to cost savings at the user level and lower investments in grid operation. Consider providing examples or case studies to illustrate potential cost savings.’* **Authors: **We have revised the description of Step 1 (iii) to better represent the impacts of reducing grid dependency. *‘Reducing dependence on the grid lowers user costs by cutting electricity purchases, as seen with residential solar installations that reduce bills. It also decreases grid investment needs by alleviating strain and maintenance requirements, minimizing the need for costly upgrades.’*  

*EM: ‘Step 2: Listing Existing and Planned Technologies: Specify how information on technologies will be collected and analyzed for sector coupling.’*

**Authors: **We have revised the description of this step to now include an overview on the data collection. *‘Data is gathered directly from project pilots through detailed asset inventories and technical specifications, ensuring accurate information on system components and energy flows.’*  

*EM: ‘Step 3: Planning Actions at Different EC Levels: Please, provide concrete examples of actions at each level to help readers understand the practical implications of data collection at the user level, integration at the EC level, and aggregation at the federation level.’*

**Authors: **We have revised this step to broaden the planned actions at each level with examples. *‘At the user level, actions include collecting data from smart meters to optimize energy usage and implementing demand-side management strategies. At the EC level, integration involves developing a cloud platform for analytics and optimizing energy use within a single EC. At the federation level, actions focus on aggregating multiple communities for intercommunity energy exchange and balancing. Detailed examples of these actions can be found in the planned actions section.’*  

*EM: ‘Clarify what "PEM electrolyser" stands for upon first mention for readers unfamiliar with the term (Proton Exchange Membrane electrolyser).’*

**Authors: **We have mentioned the full form of the term accordingly. ‘ *These can delay the installation of the proton exchange membrane (PEM) electrolyser…*’  

*EM: ‘Conclude the section “Challenges” by summarizing the key challenges and potential solutions discussed in the pilots. This will reinforce the main takeaways for the reader.’* **Authors: **We have added a conclusion paragraph to the “Challenges” section and mentioned the key challenges and possible solutions. ‘ *The implementation of clean energy scenarios in the pilots brings with it diverse challenges such as availability of installation units, regulatory hurdles, existing market rules, and user interest to participate. Mitigating these challenges involve strategies of early planning and engagement with suppliers and drawing up PPAs for favourable terms and prices. In addition, utilising digital tools, smart meters, and existing grid infrastructure, while encouraging user participation through incentive mechanisms and feed-in tariffs, play a pivotal role in a successful operation of energy communities. Advancing the clean energy transition requires for a multi-sectoral approach in planning, collaboration and building up regulatory frameworks.*’
